# *mcr-1*–Positive Colistin-Resistant *Escherichia coli* in Traveler Returning to Canada from China

**DOI:** 10.3201/eid2209.160177

**Published:** 2016-09

**Authors:** Michael Payne, Matthew A. Croxen, Tracy D. Lee, Brian Mayson, Sylvie Champagne, Victor Leung, Sherri Bariso, Linda Hoang, Christopher Lowe

**Affiliations:** Providence Health Care, Vancouver, British Columbia, Canada (M. Payne, B. Mayson, S. Champagne, V. Leung, S. Bariso, C. Lowe);; The University of British Columbia, Vancouver (M. Payne, B. Mayson, S. Champagne, V. Leung, S. Bariso, L. Hoang, C. Lowe);; British Columbia Centre for Disease Control Public Health Laboratory, Vancouver (M. Croxen, T.D. Lee, L. Hoang)

**Keywords:** *Escherichia* *coli*, colistin, antimicrobial resistance, microbial, urinary tract infection, China, Canada, MCR-1, travel-related infections

**To the Editor:** A 61-year-old man underwent transurethral prostate resection in Vancouver, British Columbia, in January 2016. On postoperative day 1, he was febrile (39.1**°**C) and had leukocytosis (12.7 × 10^9^ cells/L). Blood and urine cultures were ordered on postoperative day 2, and ceftriaxone was started. On postoperative day 3, urine culture grew *Escherichia coli* (>100 million CFU/L). Susceptibility testing (VITEK2, bioMérieux, Quebec, Canada) indicated a possible extended-spectrum β-lactamase producer and showed resistance to ampicillin, cefazolin, ceftriaxone, gentamicin, ciprofloxacin, and trimethoprim/sulfamethoxazole; intermediate resistance to tobramycin; and susceptibility to amoxicillin/clavulanate, piperacillin/tazobactam, ertapenem, meropenem, and nitrofurantoin. Treatment was switched to amoxicillin/clavulanate. The urinary catheter was removed 48 hours later. The patient was discharged on postoperative day 5 and completed 14 days of oral amoxicillin/clavulanate. Blood cultures were negative after 7 days’ incubation.

The *E. coli* cultured from the patient underwent further testing and grew in equal amounts on Columbia Colistin-Nalidixic acid Agar (CNA) with 5% sheep blood and Columbia agar with 5% sheep blood (OXOID, Ontario, Canada). This result was brought to the attention of the hospital’s medical microbiologist. A colistin Etest (bioMérieux, Quebec, Canada) showed a MIC of 3 μg/mL; EUCAST defines colistin resistance as >2 μg/mL for *Enterobacteriaceae* (*1*).

A real-time PCR to detect the mobile colistin resistance (*mcr-1*) gene was developed at the Provincial Public Health Laboratory by using primers MCR-1F (5′-CATCGCTCAAAGTATCCAGTGG-3′), MCR-1R (5′-CCATGTAGATAGACACCGTTCTCAC-3′), and probe MCR-1P (5′-Cy5-TGCAGACGCACAGCAATGCCTATGAT-TAO-3′) with TaqMan Fast Advanced Master Mix (Life Technologies, Burlington, Ontario, Canada), on an ABI 7500 FAST thermocycler (Applied Biosystems, Foster City, CA) by using manufacturer's’ recommended conditions. The *mcr-1* gene was confirmed by Sanger sequencing by using previously described oligonucleotides (*2*). The isolate was also PCR-positive for a *bla*_CTX-M_ gene. The strain was sequenced by using MiSeq (Illumina, Victoria, British Columbia, Canada), and predicted to be sequence type 3944 based on multilocus sequence typing databases (http://github.com/tseemann/mlst; http://mlst.warwick.ac.uk/mlst/dbs/Ecoli) and serotype O159:H4 (*3*). Sequence type 3944 does not belong to any clonal groups; 1 isolate from Asia is in the MLST database. Abricate (http://github.com/tseemann/abricate) and PlasmidFinder (*4*) were used to query the SPAdes-assembled genome (*5*). Results showed that this isolate carries 3 plasmids that have IncR, IncFIA/HI, and IncI2 replicons. The *mcr*-1 gene was found on a 60,599-nt contig with the IncI2 replicon; this contig is ≈87% identical to pHNSHP45 (Figure) (*2*). The *bla*_CTX-M-27_ gene was found on the same contig as the IncFIA/HI replicon, and no resistance genes were found with the IncR replicon.

**Figure:**
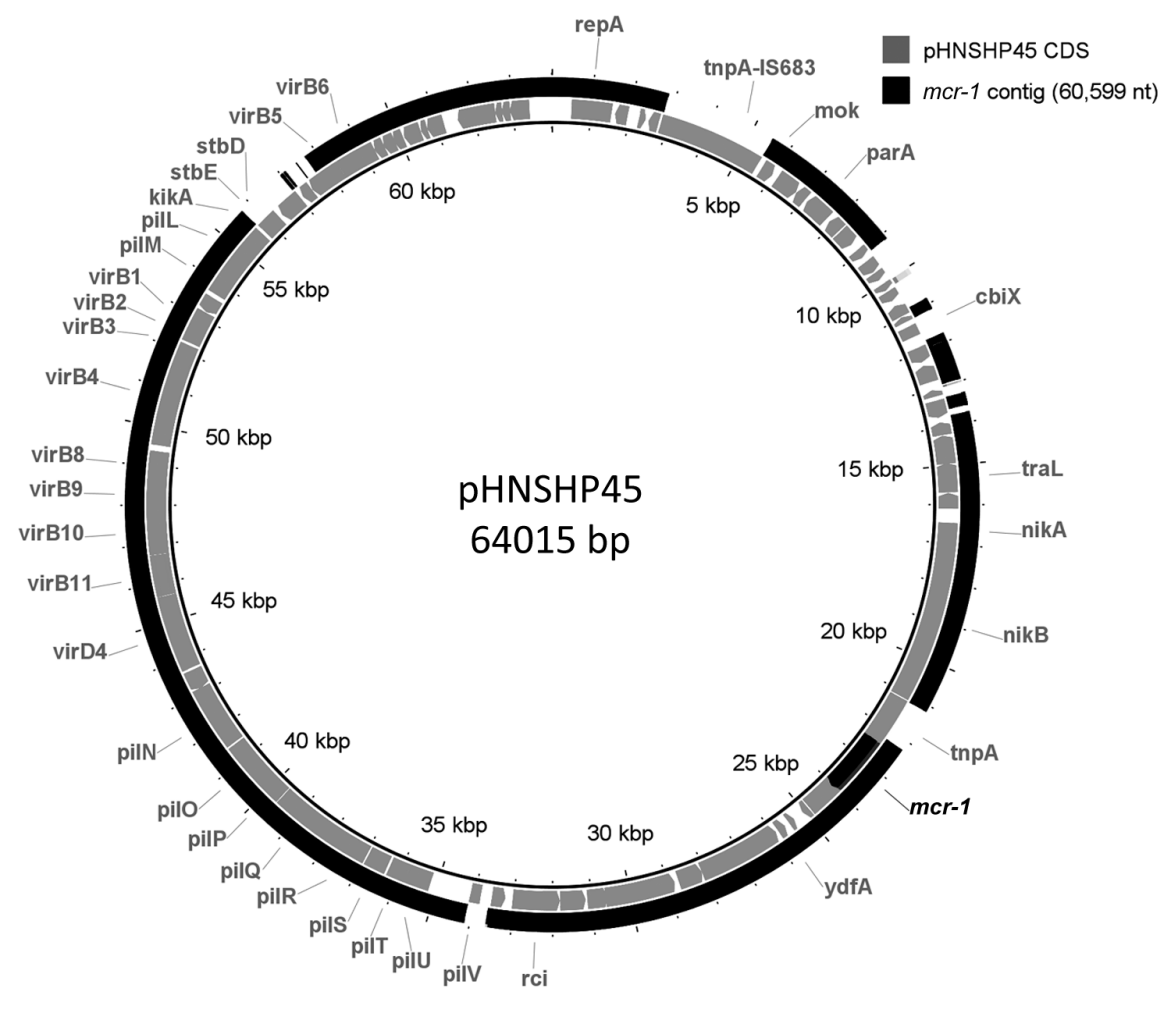
The *mcr-1*–containing contig from *Escherichia* *coli* isolated from a traveler returning to Canada from China compared to pHNSHP45 plasmid. BRIG (*6*) was used to generate a visual representation of the 60,599-nt contig assembled from the sequencing of the *mcr-1*–positive isolate to pHNSHP45 (*2*). Coding regions are represented in the inner ring; the *mcr-1*–containing contig is represented in the outer ring. The *mcr-1* gene is indicated in a black segment in the inner ring.

At the time of *mcr-1* detection, the patient and all other patients who shared a hospital room with this patient, all for <24 h, had been discharged. According to a public health representative, and on the basis of the limited exposure duration of roommates, discharged roommates were not screened. The patient improved clinically, so no changes to therapy were indicated.

The patient had traveled to China in November 2015 for 2 weeks, where he required catheterization in a hospital emergency department in Zhejiang Province for acute urinary retention. He experienced acute urinary retention and fever 6 days after catheter removal, requiring another catheter insertion and 3 days of intravenous antimicrobial drugs in Guangdong Province. He denied contact with farm animals, live poultry markets, or undercooked meat. On return to Canada, obstructive urinary tract symptoms persisted, requiring 5 emergency department visits before prostate resection. 

Colistin is a last-resort antibacterial drug because of its toxic effects and is increasingly used for treating carbapenem-resistant *Enterobacteriaceae* (CRE). Plasmid-mediated resistance genes have been described in agricultural animals and meat, as well as in humans. Initial reports described the *mcr-1* gene in China and Southeast Asia (*2**,**7*). Retrospective reviews have detected *mcr-1* in *Enterobacteriaceae* from Europe, South America, Africa, and Japan (*7*).

Unlike laboratory detection of CRE, where screening media and automated susceptibility panels were available, no commercial screening media exist for *mcr-1*. MIC testing is only recommended for *Enterobacteriaceae* resistant to all other antimicrobial classes, and molecular testing may not be accessible. Furthermore, some *Enterobacteriaceae* are intrinsically resistant to colistin. Only Etest (http://etest.net/) could be performed in our laboratory, which is a limitation that may underestimate the actual MIC (*8*). However, the reference broth microdilution method is unavailable to most clinical laboratories. This isolate was identified by a technologist who recognized heavy growth of *E. coli* on a CNA plate, an unusual occurrence because CNA plates are used for the isolation of gram-positive bacteria while inhibiting gram-negative bacteria. Despite serendipitously identifying *mcr-1* on the CNA, this method is an inadequate for detection of *mcr-1*.

Retrospective screening has identified *mcr-1* isolates in Canada (*9*). However, we describe a prospectively identified patient in Canada with *E. coli* harboring the *mcr*-*1* gene. The patient’s travel history suggested that acquisition occurred in China, although only 1% of inpatients with infection in Guangdong/Zhejiang Provinces harbor *mcr-1* (*2*).

Limited laboratory screening procedures have implications for laboratories and public health. Routine colistin testing for *Enterobacteriaceae* would be costly and low-yield; however, without such testing, the real prevalence of *mcr-1* will be underestimated. A coordinated approach to the prevention of *mcr-1* dissemination is needed, particularly to prevent the proliferation of an organism harboring a plasmid with *mcr-1* and a carbapenemase (*10*).
